# ^66^Ga: A Novelty or a Valuable Preclinical Screening Tool for the Design of Targeted Radiopharmaceuticals?

**DOI:** 10.3390/molecules23102575

**Published:** 2018-10-09

**Authors:** Alejandro Amor-Coarasa, James M. Kelly, Shashikanth Ponnala, Anastasia Nikolopoulou, Clarence Williams, John W. Babich

**Affiliations:** 1 Division of Radiopharmaceutical Sciences and MI3, Department of Radiology, Weill Cornell Medicine, New York, NY 10065, USA; ala2041@med.cornell.edu (A.A.-C.); jak2046@med.cornell.edu (J.M.K.); shp2018@med.cornell.edu (S.P.); ann2010@med.cornell.edu (A.N.); clw2012@med.cornell.edu (C.W.J.); 2 Citigroup Biomedical Imaging Center, Weill Cornell Medicine, New York, NY 10065, USA; 3 Sandra and Edward Meyer Cancer Center, Weill Cornell Medicine, New York, NY 10065, USA

**Keywords:** ^66^Ga, targeted radiotherapy, preclinical screening, PET imaging

## Abstract

Emerging interest in extending the plasma half-life of small molecule radioligands warrants a consideration of the appropriate radionuclide for PET imaging at longer time points (>8 h). Among candidate positron-emitting radionuclides, ^66^Ga (t_1/2_ = 9.5 h, β+ = 57%) has suitable nuclear and chemical properties for the labeling and PET imaging of radioligands of this profile. We investigated the value of ^66^Ga to preclinical screening and the evaluation of albumin-binding PSMA-targeting small molecules. ^66^Ga was produced by irradiation of a ^nat^Zn target. ^66^Ga^3+^ ions were separated from Zn^2+^ ions by an optimized UTEVA anion exchange column that retained 99.99987% of Zn^2+^ ions and allowed 90.2 ± 2.8% recovery of ^66^Ga^3+^. Three ligands were radiolabeled in 46.4 ± 20.5%; radiochemical yield and >90% radiochemical purity. Molar activity was 632 ± 380 MBq/µmol. Uptake in the tumor and kidneys at 1, 3, 6, and 24 h p.i. was determined by µPET/CT imaging and more completely predicted the distribution kinetics than uptake of the [^68^Ga]Ga-labeled ligands did. Although there are multiple challenges to the use of ^66^Ga for clinical PET imaging, it can be a valuable research tool for ligand screening and preclinical imaging beyond 24 h.

## 1. Introduction

Molecular imaging techniques such as CT, PET, and SPECT have accelerated the drug discovery process by reducing the time, and sometimes costs, needed to screen candidate compounds [1,2]. Preclinical µPET imaging can be used to confirm targeting in vivo and eliminate compounds with a poor stability and/or pharmacokinetics, thereby minimizing the unjustified use of costly animal models. ^68^Ga (t_1/2_ = 67 min, β^+^ = 89%) is particularly well-suited to PET imaging studies with small molecules and peptides as its half-life is well-matched to their fast blood clearance and target localization [3]. However, there is emerging interest in extending the plasma residence time of targeted radioligand therapeutics in order to increase tumor loading and enhance the anti-tumor effect [4,5,6,7,8,9]. The short half-life of ^68^Ga renders it less suitable for informative imaging of these ligands. To properly screen these radioligands, radionuclides with a physical half-life that more closely matches the longer biological half-life of the ligand are required.

^89^Zr (t_1/2_ = 78.4 h, β^+^ = 23%) is a long-lived isotope suitable for the PET imaging of vectors with long circulation half-lives, such as antibodies or nanoparticles [10,11]. Although the longer half-life is advantageous in this context, it may be excessive for smaller molecules or peptides with faster kinetics and therefore associated with unjustified levels of radiation exposure. ^64^Cu (t_1/2_ = 12.7 h, β^+^ = 18%) is a shorter half-life alternative that has been used to image with a high resolution both smaller molecules and peptides with faster distribution kinetics [12,13], as well as antibodies with a longer circulation time [14]. However, the co-emission of a β^-^ particle (38%) increases radiation exposure and the chelation of ^64^Cu by DOTA is not optimally stable [15,16]. ^86^Y (t_1/2_ = 14.7 h, β^+^ = 33%) has been used to radiolabel DOTA-containing small molecule or peptide tracers [17,18,19,20]. Despite ongoing optimization of cyclotron-based production methods that deliver ^86^Y in high starting activities and high radionuclidic purity [21,22], the high positron energy and significant gamma emissions associated with ^86^Y decay confound imaging and quantification [23,24]. More recently, ^44^Sc (t_1/2_ = 4.0 h, β^+^ = 94%) has been conjugated to small molecules and/or peptides [25,26,27,28] by highly stable complexation with DOTA. However, although starting activities of up to 350–370 MBq have been achieved from proton irradiation of an enriched ^44^Ca [27] or a natural ^nat^Ca target [26], high quality imaging using ^44^Sc may be limited to 20–24 h post injection and so unsuitable for those radioligands that still exhibit significant blood pool activity at this time point.

^66^Ga (t_1/2_ = 9.5 h, β^+^ = 57%) has been used as an alternative to ^68^Ga for PET imaging of radioligands with slower tissue distribution [29,30,31]. Batches of up to 7.4 GBq at end-of-bombardment have been produced by the proton irradiation of ^66^Zn [30], while lower activities of ^66^Ga have been formed in comparable radionuclidic purity from irradiation of a ^nat^Zn target [30,32]. The combination of half-life and starting activities produced has allowed ^66^Ga-labeled PET tracers to be imaged up to 36 h post injection [33]. Although the positron energy is greater than that of other PET isotopes, leading to decreases in image resolution [34,35], the resulting image quality is superior to that obtained from SPECT [30,32]. We aimed to determine the value of ^66^Ga µPET imaging to the preclinical development of a series of albumin-binding radioligands targeting PSMA in prostate cancer xenograft tumors and to evaluate its potential clinical utility in comparison to radionuclides such as ^68^Ga.

## 2. Results

### 2.1. Production and Purification of ^66^Ga

Irradiation of the ^nat^Zn target was initially performed at a 20 μA proton current for 2 h, but although the activity of ^66^Ga achieved at end of bombardment (EOB) was 8.6 ± 0.2 GBq (Table 1), this current resulted in high target temperatures that melted the center of the target. Subsequently, the current was reduced to 17 μA, resulting in a ^66^Ga activity at EOB of 7.2 ± 1.1 GBq (Table 1). The activities of ^66^Ga and ^67^Ga at EOB were calculated by correcting for decay the activities of each radioisotope measured at the time of target processing.

At EOB, over 90% of the total activity was due to ^68^Ga, with the percentage of ^66^Ga and ^67^Ga slightly increasing with current. The target was allowed to decay overnight (20.6 ± 1.9 h, 18–23 h range), at which point the radioisotopic composition was <3.7 MBq (<0.1%) ^68^Ga, 1.67 ± 0.27 GBq (91.2 ± 1.0%) ^66^Ga, and 158.7 ± 17.5 MBq (8.8 ± 1.0%) ^67^Ga. The production yield at 17 µA was 211 ± 33 MBq/µAh, in line with previously reported yields for comparable irradiation times [30,32].

**Figure 1:**
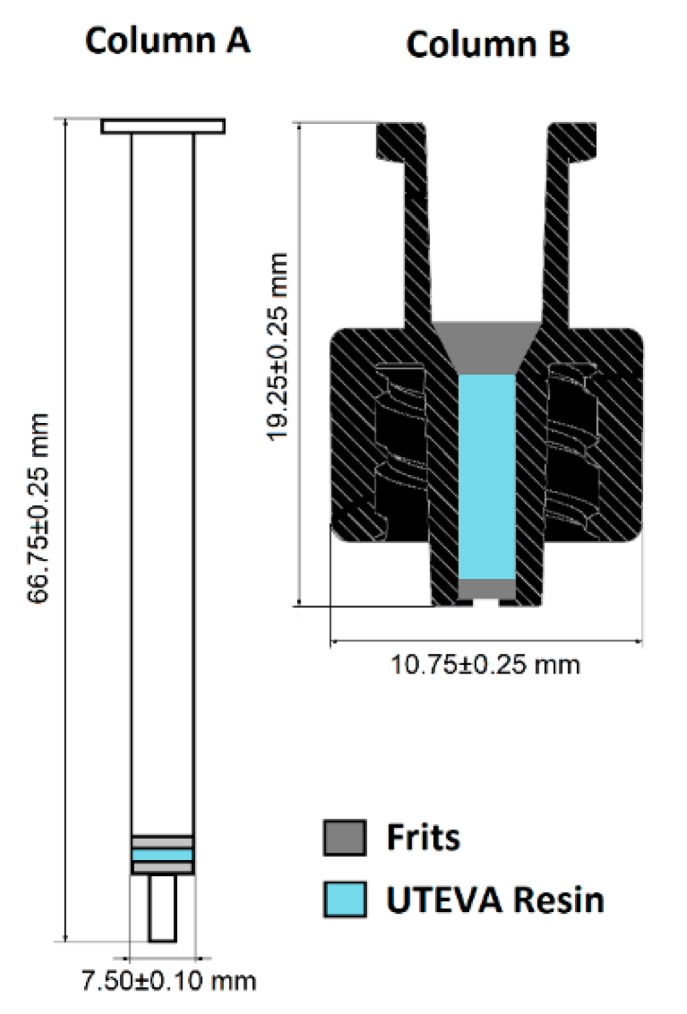
Schematic representation of the columns used for purification. Column A is a 1.5 mL, dual fritted filtration polypropylene cartridge. Column B is fabricated from a female-to-male Luer closure drilled in the male end cap with a 0.5 mm drill bit. Both columns were loaded with 20 mg of UTEVA resin as described previously [36].

The target was processed and purified in columns packed with UTEVA resin (Figure 1) according to previously published methods [36]. The trapping efficiency of column A (Figure 1) after target digestion was 98.6 ± 1.0 (n = 4), and 88.9 ± 9.6% of the trapped activity was eluted with 0.5 mL H_2_O, giving a total purification yield of 87.7 ± 10.0%. When the geometry of the UTEVA resin bed was changed to the configuration of column B (Figure 1), the ^66^Ga trapping efficiency was 95.3 ± 2.5 (n = 8). Elution of the column with 0.5 mL H_2_O yielded 94.8 ± 2.0% of the trapped activity, giving a total ^66^Ga recovery of 90.3 ± 4.1%.

### 2.2. Measurement of Metal Contaminants in the Purified ^66^Ga Solution

The eluates from column A and column B were analyzed by ICP-MS to determine the content of metal contaminants. Potentially relevant differences were seen in the Cu content (52.92 ± 1.98 ppb vs. 1.38 ± 1.77 ppb) and Al content (21.95 ± 6.73 ppb vs. 3.73 ± 4.76 ppb) in the eluates of columns A and B, respectively (Table 2). These metals are likely to be contaminants present in the Zn foil. The concentration of Fe in the eluate was nearly identical between the two column configurations. The amount of each of these metals was three orders of magnitude lower than the amount of precursor ligand, meaning that these contaminants were unlikely to influence radiolabeling yields. Zn concentration was reduced by more than 95% in the eluate of column B (12,800 ± 6100 ppb vs. 322,300 ± 21,300 ppb). Given an elution volume of 0.5 mL, this corresponds to a decrease in the mass of Zn from 161 ± 10.8 µg to 6.4 ± 3.1 µg.

### 2.3. Radiolabeling of DOTA-Containing PSMA-Targeting Ligands with Purified ^66^Ga

In spite of the high trapping and recovery of ^66^Ga, radiolabeling of the DOTA-containing compounds was achieved in consistently poor radiochemical yields (<10%; n = 6) using the elution from column A. This is likely to be due to the breakthrough of Zn^2+^ ions into the eluate (Table 2). A mass of Zn in the region of 161 ± 10.8 µg represents a stoichiometric excess with respect to the DOTA-containing precursor and an even larger excess with respect to ^66^Ga^3+^ ions. Labeling yields using the purified eluate from column B, although improved with respect to the ^66^Ga processed with column A, remained modest (46.4 ± 20.5%; n = 9). The improved yields are likely to be due to the 25-fold reduction in Zn mass in the eluate (Table 2). Nevertheless, a mass of 6.4 ± 3.1 µg of Zn^2+^ still corresponds to an important excess with respect to ^66^Ga^3+^.

One method to increase the labeling yield is to increase the mass of precursor ligand. However, we chose to perform the radiolabeling using 1.9–2.3 nmol of each precursor in order to administer a fixed mass of ligand to the mice for the imaging studies. Under these conditions, the molar activity of the final [^66^Ga]Ga-labeled compounds was 632 ± 380 MBq/µmol (range 241–1129 MBq/µmol), and the radiochemical purity was greater than 90%.

### 2.4. µPET/CT Imaging of [^66/68^Ga]RPS-063, [^66^Ga]RPS-067 and [^66/68^Ga]PSMA-617

**Figure 2:**
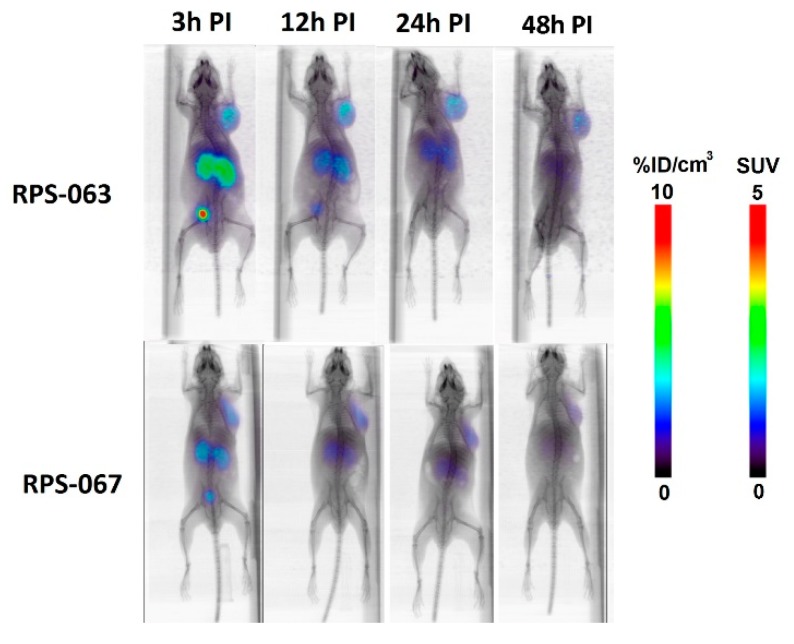
µPET/CT imaging of LNCaP xenograft tumor-bearing mice with [^66^Ga]RPS-063 and [^66^Ga]RPS-067 at 3 h, 12 h, 24 h, and 48 h post injection. Mice were injected intravenously with a bolus injection of 1.1 MBq ([^66^Ga]RPS-063 or 5.4 MBq ([^66^Ga]RPS-067). The total amount of ligand injected was 2.1 ± 0.2 nmol. Prior to imaging, the mice were anesthetized with isoflurane and then imaged for 30 min (3 h, 12 h) or 60 min (24 h, 48 h). The images were corrected for decay and for activity injected.

As an initial proof of concept, [^66^Ga]RPS-063 and [^66^Ga]RPS-067 were prepared and their distribution in LNCaP xenograft tumor-bearing mice compared. Uptake of [^177^Lu]RPS-063 had previously been shown to be greater in tumors and kidneys than [^177^Lu]RPS-067 [9]. Uptake of the [^66^Ga]Ga-labeled tracers at 3 h post injection (p.i.) was evident in the kidney and tumor (Figure 2). Urinary clearance to the bladder was the predominant route of excretion. Residual blood activity was evident for [^66^Ga]RPS-063 along with accumulation in the liver, although this liver activity was not evident for [^177^Lu]RPS-063 [9]. By 12 h p.i., the signal was concentrated in the tumor, kidneys, and bladder. The greater uptake in both the tumor and kidneys was observed for [^66^Ga]RPS-063, which remained at 7.7 ± 2.8%ID/cm^3^ and 1.3 ± 0.7%ID/cm^3^, respectively, at 24 h p.i. In contrast, the peak uptake of [^66^Ga]RPS-067 in the tumor (5.5 ± 2.6%ID/cm^3^) and kidneys (1.5 ± 0.3%ID/cm^3^) was substantially lower, and clearance by 24 h was also pronounced. These trends were consistent with the relationships determined by biodistribution studies with the [^177^Lu]Lu-labeled compounds [9].

**Figure 3:**
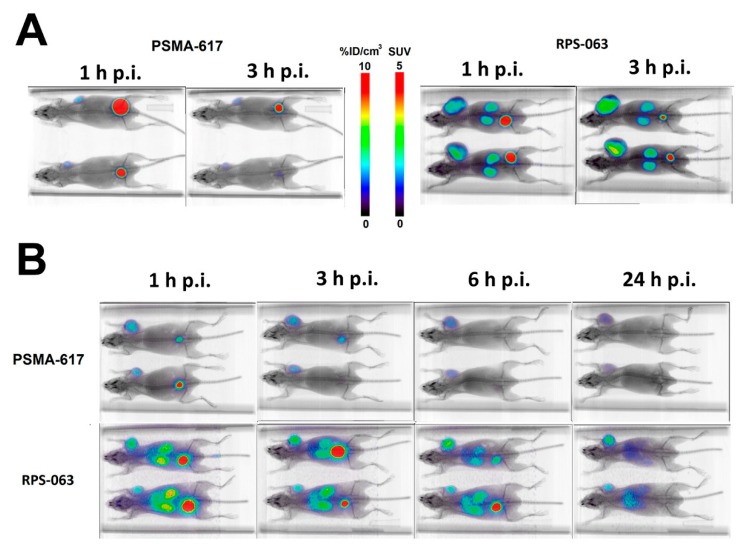
Comparison of µPET/CT images of [^66/68^Ga]PSMA-617 and [^66/68^Ga]RPS-063 in LNCaP xenograft tumor-bearing mice. Mice were injected with a bolus injection of (**A**) 8.9–11.1 MBq [^68^Ga]PSMA-617 or [^68^Ga]RPS-063, or (**B**) 1.3–1.7 MBq [^66^Ga]PSMA-617 or [^66^Ga]RPS-063, and imaged under isoflurane for 30 min (1 h, 3 h, and 6 h) or 60 min (24 h).

On the basis of its more promising biodistribution, RPS-063 was labeled with ^66^Ga or ^68^Ga and compared to [^66/68^Ga]PSMA-617. Greater uptake of [^66/68^Ga]RPS-063 was observed in the tumor and kidneys than the [^66/68^Ga]PSMA-617 counterparts at all time points (Figure 3). The absence of [^66/68^Ga]PSMA-617 in the kidneys at 1 h p.i. is surprising given previous reports [37], but the molar activity of [^68^Ga]PSMA-617 (3.28 GBq/µmol) was up to 50-fold lower than that of the [^68^Ga]PSMA-617 used in those studies. It is possible that tracer binding to PSMA in the kidney is therefore blocked by the cold ligand, whose affinity for PSMA is comparable to that of Ga-PSMA-617 [37]. The molar activity of [^68^Ga]PSMA-617 and [^68^Ga]RPS-063 was 3.28–3.44 GBq/µmol, approximately five-fold greater than the molar activity of [^66^Ga]PSMA-617 and [^66^Ga]RPS-063.

**Figure 4:**
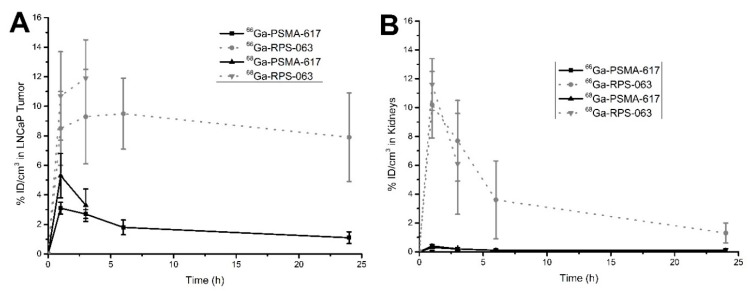
Time-activity curves of [^66/68^Ga]PSMA-617 and [^66/68^Ga]RPS-063 in LNCaP xenograft tumors (**A**) and kidneys (**B**). The activities were derived from µPET/CT images by defining a VOI and comparing the corresponding counts to a standard of known activity.

Time-activity curves (TACs) were derived for [^66/68^Ga]PSMA-617 and [^66/68^Ga]RPS-063 in tumors and kidneys (Figure 4). Up to 3 h p.i., the TACs of the [^68^Ga]Ga-labeled ligands were not significantly different to those of the [^66^Ga]Ga-labeled ligands, but when the ^68^Ga TACs were extrapolated, they diverged from the ^66^Ga curves in an important way. The activity of [^66^Ga]RPS-063 in tumors closely matched the tumor kinetics reported for [^177^Lu]RPS-063, for which tumor uptake peaked at 4 h p.i. and slightly decreased to 24 h p.i. [9]. In contrast, if the [^68^Ga]RPS-063 curve in the tumor was to be extrapolated to 24 h p.i., it would incorrectly predict a higher tumor accumulation than was observed due to its inability to capture the clearance phase. Due to the rapid distribution kinetics of PSMA-617, which exhibits fast clearance from blood [9], [^68^Ga]PSMA-617 underestimates the tumor uptake over the 24 h period.

The short half-life of ^68^Ga causes [^68^Ga]RPS-063 to underestimate the dose to the kidney. Two clearance phases are evident: an initial rapid phase and then a slower second phase (Figure 4). The second phase is only evident after 3 h p.i., and therefore extrapolation of the [^68^Ga]RPS-063 curve to 24 h p.i. would predict more rapid clearance than is observed. The shape of the [^66^Ga]RPS-063 is once again consistent with that reported for [^177^Lu]RPS-063 [9]. As the uptake of [^66/68^Ga]PSMA-617 in kidneys was low in this study, the two time-activity curves are virtually indistinguishable.

## 3. Discussion

Although 6.4 µg of Zn^2+^ was detected in the ^66^Ga-containing elution from an optimized column, and was likely responsible for the low and variable labeling yields, given a starting mass of Zn foil of 0.5 g, purification was 99.99987% efficient. The reduction in Zn breakthrough corresponded to an increase in the molar activity of the [^66^Ga]Ga-labeled products, 632 ± 380 MBq/µmol. Previously, a maximum molar activity of 370 MBq/µmol was reported using ^66^Ga obtained from irradiation of a 250 µm ^nat^Zn foil target [32]. Contamination by Fe in the foil and Zn from the mass of foil used were hypothesized to limit molar activity [32]. Using column B as purification apparatus, we were able to reduce the trapping of contaminant cations by optimizing the geometry of the column, the surface area of the column bed, and the solvent volume used. This enabled us to increase the molar activities of our radiotracers. Further improvements in the separation and purification process could ultimately be achieved by techniques such as thermal diffusion [38].

The comparison of ^66^Ga and ^68^Ga, by keeping the metal constant, should preserve the affinity and/or pharmacokinetics of the ligands investigated. Therefore, differences in the TACs up to 3 h p.i. are likely to be due to differences in the molar activity of the tracers. The molar activity of [^68^Ga]RPS-063 and [^68^Ga]PSMA-617 was approximately five-fold higher than that of their ^66^Ga counterparts, which appears to lead to slightly higher tumor uptake at early time points. Interestingly, the difference in kidneys is less pronounced. As ^68^Ga imaging could not be performed after 3 h p.i., it could not be determined whether the TACs would converge at longer time points. The effect of molar activity on tumor uptake could be studied by varying the amount of ligand added to the [^68^Ga]Ga-labeled tracers, but this experiment is beyond the scope of this work.

Notwithstanding the relatively low molar activity of the [^66^Ga]Ga-labeled radioligands, their rapid clearance from the mice, and the high positron maximum energy (E(β^+^)_max_ = 4.153 MeV) of the ^66^Ga emissions, the µPET image quality was high, even up to 48 h. Although the image quality is less clear than that obtained from ^68^Ga or ^18^F in the same camera, and depends on the pharmacokinetics of the radioligand under investigation, structures including kidneys and tumors could be clearly delineated and tracer uptake quantified. To our knowledge, PET imaging of a [^66^Ga]Ga-labeled tracer has not been performed beyond 36 h p.i. to date [33], but these studies, albeit preliminary, demonstrate the potential for ^66^Ga imaging up to 48 h p.i.

The objective of preclinical screening is to acquire the greatest information at the lowest cost. Factors that contribute to information acquisition include the physical properties of the radionuclide, which influence the image resolution and imaging time points, and the chemical properties of the radionuclide, such as its chelation chemistry and molar activity, which affect the design and pharmacokinetics of the radiotracer. Costs include the price of the target and/or generator required to produce the radionuclide, the radiation exposure introduced during the purification and imaging process, and the requirement for storage of longer-lived radionuclide by-products prior to disposal.

A list of common PET radionuclides is found in Table 3. The isotopes in group A, including ^68^Ga, have short half-lives that preclude accurate screening for ligands whose distribution and clearance are not completed within 3–4 h, such as the albumin-binding radioligand RPS-063. Among the isotopes listed in Group B, ^89^Zr and ^52^Mn have half-lives longer than three days, which is typically a better match for immuno-PET than for screening small molecule or peptide radioligands. The co-emission of high energy gamma particles, either by these radionuclides or by inseparable by-products such as ^54^Mn (t_1/2_ = 312.3 d), may increase radiation exposure above a level that preclinical screening justifies. The radiohalides ^124^I and ^79^Br must be installed via a covalent bond, and so their chemistry may not be suitable for the screening of ligands such as PSMA-617 and RPS-063.

Given these considerations, ^44^Sc, ^66^Ga, ^64^Cu, and ^86^Y (Table 3) are perhaps the most versatile and valuable radionuclides for the preclinical screening of targeted small molecule radioligands. Each has a half-life well-matched to the biological half-life of the majority of radioligands of this class, and all can be chelated by bifunctional DOTA macrocycles. However, ^64^Cu and ^86^Y are produced by the irradiation of enriched ^64^Ni and ^86^Sr targets, respectively, and the high cost of these targets necessitates a target recycling strategy [21,39]. In contrast, ^44^Sc and ^66^Ga may be produced from ^nat^Ca or ^nat^Zn targets, respectively, leading to relatively high activities in high radionuclidic purity. The principle long-lived impurity formed in each production, ^47^Sc (t_1/2_ = 80 h) and ^67^Ga (t_1/2_ = 78 h), is less than 3% of the total purified activity and does not represent an unreasonable burden in terms of exposure or waste management. The high energy positron and gamma co-emissions of ^66^Ga (β^+^ = 57%) elevate radiation exposure [40] relative to ^44^Sc (β^+^ = 94%). Ultimately, however, the shorter half-life of ^44^Sc may prove limiting if radioligands with slower clearance such as PSMA-Alb-53 or PSMA-Alb-56 [7] are screened. These ligands have shown continued accumulation in tumors beyond 24 h, at which point images with ^44^Sc might be expected to decrease in quality.

## 4. Materials and Methods

### 4.1. Synthesis of Precursors and Ligands

**Figure 5:**
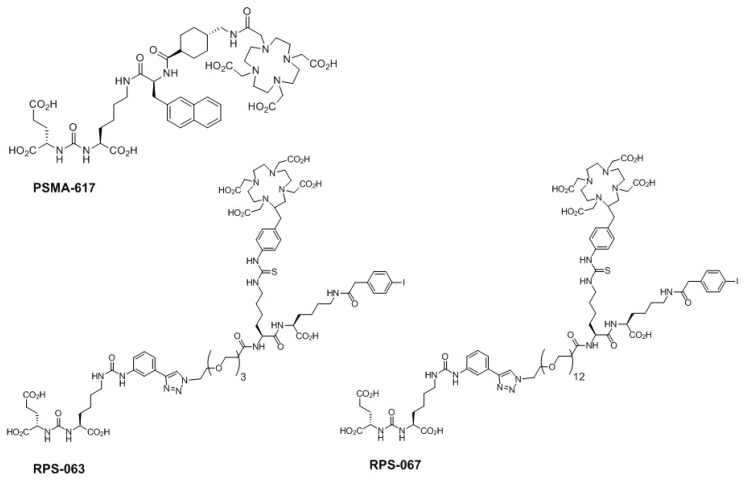
Structures of PSMA-617, RPS-063, and RPS-067.

PSMA-617, the precursor for [^68^Ga/^177^Lu/^90^Y]PSMA-617, was purchased from ABX and used without further purification. RPS-063 and RPS-067 were prepared as previously described [9], and the structures are shown in Figure 5.

### 4.2. Radiochemistry

#### 4.2.1. General Methods

All reagents were purchased from Sigma Aldrich unless otherwise noted, and were reagent grade. Hydrochloric acid (HCl) and sodium acetate (NaOAc) were of traceSELECT^®^ (>99.999%) quality. All water (H_2_O) used was highly pure (18 mΩ). Analytical HPLC was performed on a dual-pump Varian Dynamax HPLC (Agilent Technologies, Santa Clara, CA, USA) fitted with a dual UV-Vis detector, and radiochemical purity was determined using a NaI(Tl) flow count detector (Eckert & Ziegler Radiopharma, Inc., Hopkinton, MA, USA). UV absorption was monitored at 220 nm and 280 nm. Solvent A was 0.01% trifluoroacetic acid (TFA) in H_2_O and solvent B was 0.01% TFA in 90% *v*/*v* acetonitrile (MeCN):H_2_O. Analyses of the [^66/68^Ga]Ga-labeled products were performed on a Symmetry C18 5 µm, 4.6 × 50 mm, 100 Å column (Waters, Milford, MS, USA) at a flow rate of 2 mL/min and a gradient of 0%B to 100%B over 5 min.

#### 4.2.2. Production of ^66^Ga

Gallium-66 (t_1/2_ = 9.4 h) was produced from the irradiation of a natural zinc (^nat^Zn) foil target (Alfa Aesar; 0.5 g, 100 µm thickness, 99.999%) by a (p,n) reaction over 2 h using a 15 MeV beam and a 17 µA current (n = 6). The irradiation of natural zinc produced ^66^Ga, ^67^Ga, and ^68^Ga. The target was left to decay overnight (20.6 ± 1.9 h, 18–23 h range) to allow ^68^Ga (t_1/2_ = 68 min) to decay before processing. The principal radionuclidic impurity during processing was ^67^Ga (t_1/2_ = 78.3 h). The target was dissolved in conc. HCl (12 M, 5 mL) and the ^66^Ga^3+^ ions were separated from Zn^2+^ ions over 20 mg UTEVA anion exchange resin (Eichrom, IL, USA) packed in two different configurations: a 1.5 mL polypropylene filter cartridge (Agilent) or a micro column constructed according to previously published methods [36]. The column was washed twice with HCl (5 M, 3 mL) to eliminate the excess Zn^2+^. Finally, the purified ^66^Ga^3+^ ions were eluted with H_2_O (0.5 mL), leading to a stock solution of ^66^Ga in approximately 0.1 M HCl.

#### 4.2.3. Radiolabeling of PSMA-617, RPS-063, and RPS-067

The [^66^Ga]Ga-labeled ligands were prepared by the addition of 40 µL of a 1 mg/mL solution of RPS-063 or RPS-067 in DMSO to 50 µL of the ^66^Ga stock solution containing 167–205 MBq. The radiolabeling of PSMA-617 was performed using 30 µL of a 0.75 mg/mL solution of precursor in DMSO. The reaction was initiated by the addition of 10 µL 3 N NaOAc, and the solution was mixed at 95 °C on an Eppendorf ThermoMixer^®^ C (VWR, Radnor, PA, USA) for 15 min. The mixture was then diluted with H_2_O and passed through a pre-activated Sep-Pak™ C18 Plus Light cartridge (Waters, Milford, MA, USA). The cartridge was washed with H_2_O and the product was eluted with 200 µL of a 50% *v*/*v* EtOH (200 proof, VWR)/saline (0.9% NaCl solution; VWR) solution, followed by 800 µL saline.

[^68^Ga]RPS-063 and [^68^Ga]PSMA-617 were labeled by the same procedure, with small modifications. [^68^Ga]GaCl_3_ (198 MBq) was eluted from a 50 mCi ^68^Ge/^68^Ga generator (ITG GmbH, Garching, Germany) in 4 mL of a 0.05 M HCl solution. To this stock solution, 40 µL of a 1 mg/mL solution of precursor in DMSO was added. The pH was adjusted to 4–5 by the addition of 80 µL 3 N NaOAc, and the reaction was heated for 15 min at 95 °C on an Eppendorf ThermoMixer^®^ C (VWR, Radnor, PA, USA). The purification was performed as described above.

### 4.3. Sample Analysis by ICP-MS

Non-radioactive samples of the dissolved target and the elution fractions were analyzed by ICP-MS at the Department of Earth and Environmental Sciences at Brooklyn College of The City University of New York. The 0.5 mL eluate from the UTEVA column (each configuration) was diluted to 1/20 in 1% HNO_3_ (TraceSelect for trace metal analysis, Sigma Aldrich, St. Louis, MO, USA). As a control, samples were prepared following the elution protocol in the absence of Zn. All experiments were performed in triplicate. The samples were then analyzed by ICP-MS, and the concentrations of Al, Cr, Co, Cu, Fe, Mn, Ni, and Zn were determined, corrected for sample dilution, and expressed as ppb (ng/mL) ± standard deviation.

### 4.4. µPET Imaging Studies in LNCaP Xenograft Tumor-Bearing Mice

#### 4.4.1. Inoculation of Mice with Xenografts

All animal studies were approved by the Institutional Animal Care and Use Committee of Weill Cornell Medicine (Protocol Number: 2015-0003) and were undertaken in accordance with the guidelines set forth by the USPHS Policy on Humane Care and Use of Laboratory Animals. Animals were housed under standard conditions in approved facilities with 12 h light/dark cycles. Food and water was provided ad libitum throughout the course of the studies. Hairless male nu/nu mice were purchased from the Jackson Laboratory (Bar Harbor, ME, USA). For inoculation in mice, LNCaP cells were suspended at 4 × 10^7^ cells/mL in a 1:1 mixture of PBS/Matrigel (BD Biosciences, San Jose, CA, USA). Each mouse was injected in the flank with 0.25 mL of the cell suspension. The mice were imaged when the tumors reached approximately 200–400 mm^3^.

#### 4.4.2. PET Imaging Studies

LNCaP xenograft tumor-bearing mice (two per compound) were injected intravenously with either a bolus injection of 2.1 ± 0.1 nmol (2–4 μg) of ligand and a total activity of [^66^Ga]Ga-labeled ligand of 1.1–5.4 MBq, or 1.5 ± 0.5 nmol (2 µg) of ligand and a total activity of [^68^Ga]Ga-labeled ligand of 8.9–11.1 MBq. The mice were imaged using μPET/CT (Inveon™; Siemens Medical Solutions, Inc., Erlangen, Germany) at 1, 3, 12, 24, and 48 h post-injection following inhalation anesthetization with isoflurane. The total acquisition time was 30 min for the 1 h, 3 h, and 12 h images, and 60 min for 24 h and 48 h time points. A CT scan was obtained immediately before the acquisition for both anatomical co-registration and attenuation correction. Images were reconstructed using the Inveon™ software supplied by the vendor. Image-derived tumor and kidney uptake were estimated by comparison to a 10% injected dose per cm^3^ (%ID/cm^3^) standard introduced into the imaging field of view. The standard was prepared by the dilution of 10% of the injected activity to 1 mL with saline. Volumes of interest (VOIs) were drawn with the aid of the CT and confirmed by PET. The contents of the VOIs were integrated and the calculated counts were converted to %ID/cm^3^ by direct comparison to the standard following correction for activity injected and decay. Data are plotted as mean ± standard deviation.

## 5. Conclusions

^66^Ga represents a reasonable compromise between information and cost. The ^nat^Zn target is inexpensive, and although our purification of ^66^Ga out of the target material could not completely remove Zn impurities, purification was 99.99987% efficient, and molar activities of the product of 632 ± 380 MBq/µmol were achieved. A large starting activity of ^66^Ga of 1.5 GBq compensated in part for modest labeling yields. The molar activities that are currently attainable limit the use of ^66^Ga for imaging systems that are sensitive to this parameter, but for PSMA-targeting ligands such as RPS-063 and RPS-067, high quality PET imaging could be achieved for as long as 48 h p.i. Imaging at longer time points enabled the kinetics of uptake and clearance in key tissues including tumors and kidneys to be better predicted than could be achieved with ^68^Ga. Clinical translation of ^66^Ga may be limited by the absence of an automated purification procedure that efficiently removes metal contaminants and reduces radiation exposure, but the radionuclide has clear value as a research tool for preclinical compound evaluation.

## Figures and Tables

**Table 1:** Comparison of ^66^Ga production following 15 MeV proton bombardment of a 100 µm ^nat^Zn foil target.

Beam Current (µA)	Irradiation Time (h)	^66^Ga Activity at EOB (GBq)	% ^66^Ga at EOB	% ^67^Ga at EOB	% ^68^Ga at EOB	^66^Ga Production Yield (MBq/µAh)
20 (n = 3)	2	8.6 ± 0.2	9.54 ± 0.22	0.24 ± 0.01	90.22 ± 0.22	215 ± 6
17 (n = 6)	2	7.2 ± 1.1	8.06 ± 1.16	0.20 ± 0.02	91.74 ± 1.16	211 ± 33

**Table 2:** The concentration of metal impurities in the eluate following the extraction and purification of ^66^Ga^3+^ ions as determined by ICP-MS. Values are expressed as ppb ± standard deviation.

Metal	Al	Co	Cr	Cu
Column A	21.95 ± 6.73	<0.10	10.94 ± 1.10	52.92 ± 1.98
Column B	3.73 ± 4.76	<0.10	< 0.77	1.38 ± 1.77
Detection Limit (ppb)	6.67	0.10	0.77	0.37
Metal	Fe	Mn	Ni	Zn
Column A	57.70 ± 10.44	<1.04	9.50 ± 0.96	322,300 ± 21,500
Column B	48.93 ± 14.33	<1.04	6.18 ± 7.43	12,800 ± 6100
Detection Limit (ppb)	20.34	1.04	0.23	0.127

**Table 3:** Common positron emitting radionuclides.

Group	Isotope	Half Life (T_1/2_)	Max 𝛃^+^ Energy (MeV)	𝛃^+^ Emission (%)	Target Material and Natural Abundance
**A**	^11^C	20.4 min	0.960	99.8	^14^N (99.6%)
	^13^N	10.0 min	1.199	100	^16^O (99.76%)
	^15^O	2.07 min	1.732	100	^15^N (0.4%)
	^18^F	109.4 min	0.635	97.0	^18^O (0.2%)
	^68^Ga	68.2 min	1.897	89.3	Gen (^68^Ge), ^68^Zn (18.5%)
**B**	^44^Sc	3.92 h	1.470	94.3	Gen (^44^Ti), ^44^Ca (2%)
	^52^Mn	5.6 days	0.575	29.6	^52^Cr (82%)
	^64^Cu	12.8 h	0.656	17.4	^64^Ni (0.9%)
	^66^Ga	9.49 h	4.153	56.5	^66^Zn (27.8%)
	^76^Br	16.2 h	3.980	57.0	^76^Se (9.1%)
	^86^Y	14.74 h	3.150	34.0	^86^Sr (9.9%)
	^89^Zr	3.27 d	0.900	22.7	^89^Y (100%)
	^124^I	4.18 d	2.130	25.0	^124^Te (4.8%)
